# *Spirulina Platensis* Supplementation Coupled to Strength Exercise Improves Redox Balance and Reduces Intestinal Contractile Reactivity in Rat Ileum

**DOI:** 10.3390/md18020089

**Published:** 2020-01-29

**Authors:** Layanne C. C. Araujo, Aline F. Brito, Iara L. L. Souza, Paula B. Ferreira, Luiz Henrique C. Vasconcelos, Alexandre S. Silva, Bagnólia A. Silva

**Affiliations:** 1 Programa de Pós-graduação em Ciências (Fisiologia Humana), Instituto de Ciências Biológicas, Universidade de São Paulo, Sao Paulo/SP 05508900, Brazil; 2 Escola de Educação Física, Universidade de Pernambuco, Recife/PE 50740-465, Brazil; alineebritoo@gmail.com; 3 Programa de Pós-graduação em Produtos Naturais e Sintéticos Bioativos, Centro de Ciências da Saúde, Universidade Federal da Paraíba, Joao Pessoa/PB, 58051900, Brazil; iara-04@hotmail.com (I.L.L.S.); paulabenvindo92@hotmail.com (P.B.F.); Henrique.luiz89@gmail.com (L.H.C.V.); 4 Departamento de Educação Física, Centro de Ciências da Saúde, Universidade Federal da Paraíba, Joao Pessoa/PB 58051900, Brazil; alexandresergiosilva@yahoo.com.br; 5 Departamento de Ciências Farmacêuticas, Centro de Ciências da Saúde, Universidade Federal da Paraíba, Joao Pessoa/PB 58051900, Brazil

**Keywords:** blue algae, *Spirulina platensis*, contractile reactivity, oxidative stress, ileum

## Abstract

The blue alga *Spirulina platensis* has presented several pharmacological activities, highlighting its actions as an anti-inflammatory and antioxidant. In addition, there are few studies with the influence of strength training on physiological parameters, as intestinal contractility and oxidative cell damage. We evaluated the influence of *S. platensis* supplementation, strength training, and its association on contractile reactivity of rat ileum, as well as the balance of oxidative stress/antioxidant defenses. Methods: Male Wistar rats were divided into; sedentary (S); S + supplemented with algae at 50 (S50), 150 (S150), and 500 mg/kg (S500); trained (T); and T + supplemented (T50, T150, and T500). Contractile reactivity was analyzed by kymographs; oxidative stress on ileum by the malondialdehyde (MDA) formation; and the antioxidant capacity by 2,2-diphenyl-1-picrylhydrazyl (DPPH) method. *S. platensis* supplementation reduced the reactivity of rat ileum to carbachol (CCh) and KCl, while training reduced only the CCh efficacy. In addition, association potentiated the reduction on contractile reactivity. Supplementation reduced the oxidative stress and increased oxidation inhibition; training alone did not alter this parameter, however association potentiated this beneficial effect. Therefore, this study demonstrated that both supplementation and its association with strength training promote beneficial effects regarding intestinal contractile reactivity and oxidative stress, providing new insights for intestinal disorders management.

## 1. Introduction

The use of natural products by humans comes from many years and science that has increasingly recognized its active action, including in modern pharmacotherapy with various drugs of vegetable origin [1,2]. Besides these products, there are those from aquatic origin, which, unlike the plants, are not commonly used in traditional medicine [3].

Despite this, several studies have reported the pharmacological importance of metabolites derived from aquatic products, such as chemotherapeutic, anti-inflammatory, hypoglycemic, and antioxidant activities [4,5,6]. In this way, aquatic bioactive products may be used as a therapeutic tool for many diseases, and therefore should be more studied for new discoveries in the field of pharmacology and therapeutics.

Some aquatic products have been already marketed and used as food supplements, such as the blue-green algae *Spirulina platensis*. It belongs to the Cyanobacterial phylum and the family Spirulinaceae [7] and has been widely used due its nutritional value. Furthermore, several studies have reported its importance for the treatment of cardiovascular, metabolic, and inflammatory diseases, in addition to its antioxidant properties [8,9,10]. Brito et al., 2018 [11] showed that chronic supplementation with *S. platensis* decreased contractile reactivity and increased relaxing activity. Also, it was shown that the factors that accompanied this improvement in reactivity involve the release of nitric oxide and reduction in oxidative stress and systemic inflammation. Accordingly, these data demonstrated for the first time that the synergistic action between strength training and *S. platensis* results in improving vascular reactivity.

Changes in intestinal contractility are responsible for several symptoms like colic, diarrhea, and constipation [12], and *S. platensis* has presented beneficial effects in gastrointestinal inflammatory diseases, such as colitis [13]. *S. platensis* was found to have beneficial effects in constipation, enhancing gut peristalsis, curing the inflammatory reaction in the chorionic villus, and modulating the composition of the intestinal microbiota of mice [14]. However, there is a lack of studies about the effect of this algae on the intestinal contractile reactivity.

Physical exercise has been recommended as a therapeutic tool for the prevention and treatment of diseases and disorders of the gastrointestinal tract. The acute and chronic aerobic swimming exercise modifies the contractile gastrointestinal reactivity, thus contributing to the improvement of these gastrointestinal symptoms [15,16]. However, nothing has been demonstrated about the influence of anaerobic exercise of resistance on intestinal contractility, and the effects of the combination of exercise and *S. platensis* supplementation.

Thus, based on the beneficial effects of *S. platensis* for health and the influence of physical exercise on organ and body systems functioning, the aim of this study was to evaluate the effect of *S. platensis* supplementation, strength exercise, and its combination, on the contractile reactivity of rat ileum.

## 2. Results

### 2.1. Electromechanical Coupling Contractile Reactivity Measurement

**Figure 1:**
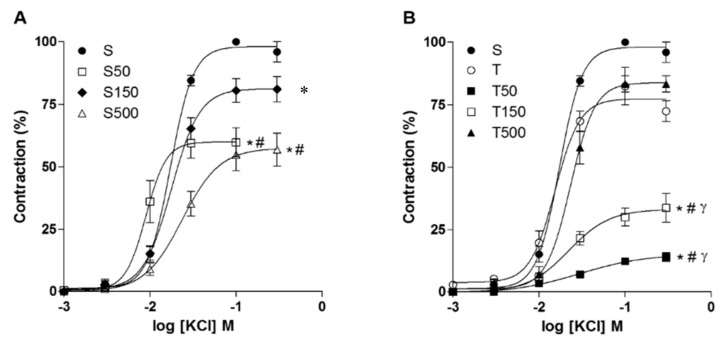
Cumulative concentration-response curves to KCl in S, S50, S150, S500 (**A**), and T, T50, T150, and T500 groups (**B**) in rat ileum. Symbols and the bars represent the mean and e.p.m., respectively (*n* = 3–5).Two-way ANOVA followed by Tukey’s post-test: * *p* < 0.05 (S vs. S50, S500, T50, and T150), ^#^ *p* < 0.05 (S50 and S500 vs. S150; T vs. T50 and T150), ^γ^ *p* < 0.05 (T50 and T150 vs. T500).

Cumulative concentration–response curves to KCl (10^−3^ to 3 × 10^−1^ M) (*n* = 3–5) were attenuated, with reduction on *E*_max_ values, by the supplementation with *S. platensis* at the doses of 50, 150, and 500 mg/kg compared to control (*p* < 0.05). The strength training did not modify the efficacy of KCl in relation to S; however, the supplementation of trained animals with the algae reduced the efficacy of KCl at doses of 50 and 150 mg/kg (*p* < 0.05), compared to S, but not at 500 mg/kg. Analysis of pEC_50_ values showed that the supplementation with the algae at 50 mg/kg increased the potency of KCl (*p* < 0.05), compared to S; however, this was not observed at doses of 150 and 500 mg/kg. In addition, the training or the association with the algae did not alter this parameter, compared to control group (Figure 1, Table 1).

### 2.2. Pharmacomechanical Coupling Contractile Reactivity Measurement

**Figure 2:**
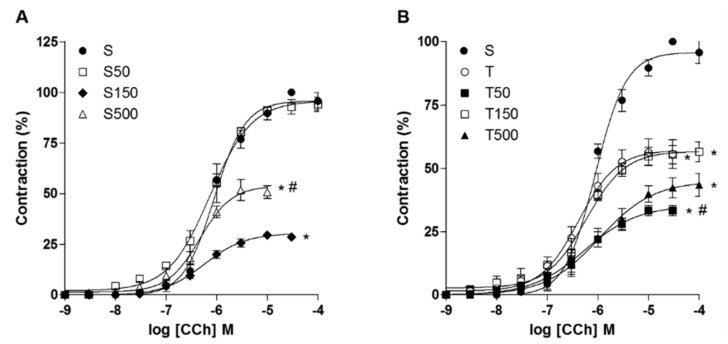
Cumulative concentration-response curves to CCh in S, S50, S150, and S500 (**A**), and T, T50, T150, and T500 groups (**B**) of rat ileum. Symbols and the bars represent the mean and e.p.m., respectively (*n* = 3–5). One-way ANOVA followed by Tukey’s post-test: * *p* < 0.05 (S or S50 vs. S150 and S500; S vs. T, T50, T150 and T500), ^#^ *p* < 0.05 (S150 vs. S500; T and T150 vs. T50).

Cumulative concentration–response curves to CCh (10^−9^ to 10^−4^ M) (*n* = 3–5) were attenuated, with the reduction of *E*_max_, by supplementation with *S. platensis* at doses of 150 and 500 mg/kg, but not at 50 mg/kg (*p* < 0.05). The strength exercise also reduced the efficacy of CCh, as well as its combination with supplementation at all doses (*p* < 0.05). Regarding the pEC_50_ values, only 500 mg/kg increased the potency of CCh, without association with training (*p* < 0.05) (Figure 2, Table 2).

### 2.3. Lipid Peroxidation Assay

**Figure 3:**
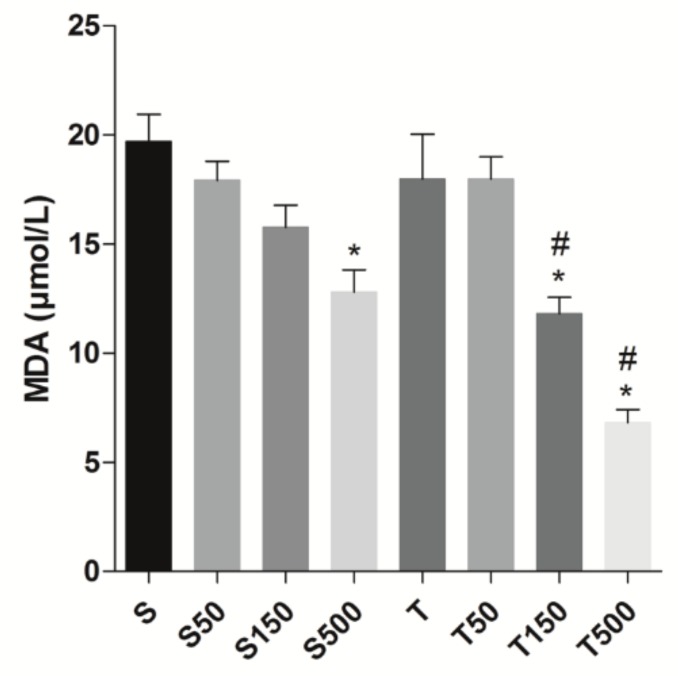
Concentration of MDA (μmol/mL) of S, S50, S150, S500, T, T50, T150, and T500 groups in rat ileum. Columns and vertical bars represent the mean ± S.E.M., respectively (*n* = 5). Two-way ANOVA followed by Bonferroni’s post-test: * *p* < 0.05 (S vs. S500; T vs. T150 and T500); ^#^ *p* < 0.05 (T50 vs. T150 and T500).

The MDA concentration in rat ileum (*n* = 5) was decreased from 19.7 ± 1.2 (S) to 12.8 ± 1.0 μmol/L by supplementation with *S. platensis* only at 500 mg/kg (*p* < 0.05), but did not differ in S50 (17.9 ± 0.0 μmol/L) and S150 (15.8 ± 1.0 μmol/L). The strength training did not alter the MDA levels in ileum 18.0 ± 2.0 μmol/L, as well as its combination with the algae at 50 mg/kg (18.0 ± 1.0 μmol/L); however, the supplementation of exercised animals at 150 and 500 mg/kg reduced the MDA concentrations to 11.8 ± 0.8 and 6.8 ± 0.6 μmol/L (Figure 3).

### 2.4. Antioxidant Assay

**Figure 4:**
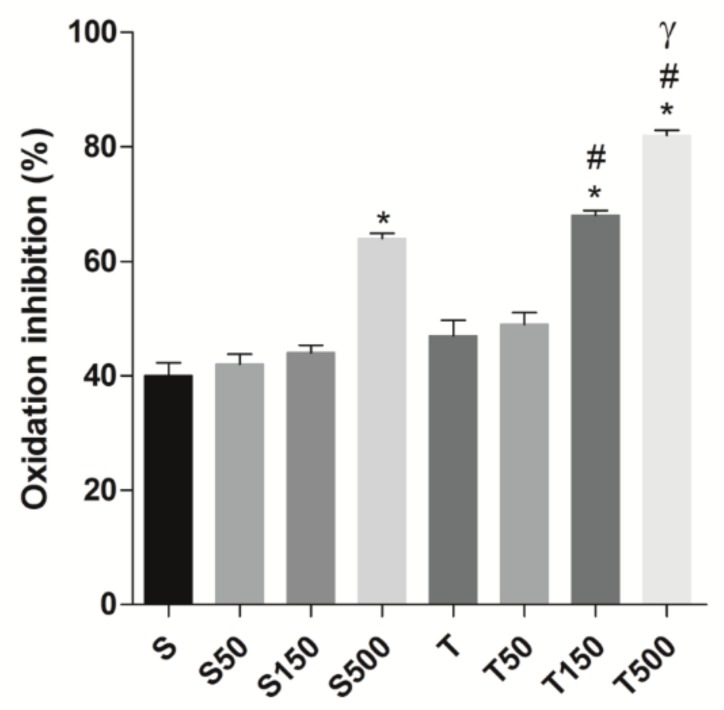
Oxidation inhibition (%) in the S, S50, S150, S500, T, T50, T150, and T500 groups in rat ileum. Columns and vertical bars represent the mean ± S.E.M. respectively (*n* = 5). Two-way ANOVA followed by Bonferroni’s post-test: * *p* < 0.05 (S vs. S500; T vs. T150 and T500); ^#^ *p* < 0.05 (T50 vs. T150 and T500); ^γ^ *p* < 0.05 (T150 vs. T500).

The oxidation inhibition, based on DPPH concentration, in rat ileum (*n* = 5) from S group (40.0 ± 5.0%) was not altered by supplementation with algae at 50 (42.0 ± 4.0%) and 150 mg/kg (44.0 ± 3.0%); but was increased to 64.0 ± 0.9% at 500 mg/kg. The strength exercise did not alter this parameter (49.0 ± 5.0%), as well as its combination with *S. platensis* supplementation at 50 mg/kg (49.0 ± 5.0%); however, at 150 (68.0 ± 2.0%) and 500 mg/kg (82.0 ± 2.0%), the supplementation associated with training increased the oxidation inhibition on rat ileum (Figure 4).

## 3. Discussion

In this work, we investigated the modulation of contractile reactivity of rat ileum by food supplementation with the blue algae *Spirulina platensis*, strength exercise and the association of supplementation with training, as well as its effects on the balance between oxidative stress and antioxidant defenses. As a result, we demonstrated that algae supplementation reduces the maximum amplitude of the intestinal contractile response to both electro- and pharmaco-mechanical stimulation, in sedentary animals or those submitted to strength exercise. However, the physical exercise alone negatively modifies only the reactivity to pharmacomechanical coupling of contraction. Additionally, although the exercise program did not promote alterations in the overall balance between reactive oxygen species (ROS) production and antioxidant defenses in the intestine, algae supplementation reduced oxidative stress as well as improved intestinal antioxidant defenses in both sedentary and trained rats.

The importance of natural products as a therapeutic choice for numerous diseases has been already described. Among these natural products, the aquatic algae *Spirulina platensis* is highlighted, having presented some pharmacological activities, such as hypoglycemic, anti-inflammatory and antioxidant, contributing to the prevention and treatment of cardiovascular, metabolic, and inflammatory diseases [12]. Indeed, studies with animals and humans have demonstrated that the supplementation with the algae prevents oxidative stress, as well as the endothelial dysfunction [17,18].

We postulated that oral supplementation with *S. platensis* could alter, in some way, the intestinal contractile reactivity, which modifies the gastrointestinal functioning. For this, CCh, a muscarinic agonist, was used as pharmacomechanical contractile agent mimicking the acetylcholine (ACh) effects, as it is released from myenteric autonomic nerves to regulate intestinal smooth muscle motility [19,20]. This agonist binds to M_3_ receptors, which leads to G_q/11_ protein pathway activation, and stimulation of phospholipase C, resulting on Ca^2+^ influx and release from sarcoplasmic reticulum and, then, intestinal muscle contraction [21,22,23,24]. Additionally, to simulate the electric pacemaker of interstitial cells of Cajal located at the boundaries and in the substance of the inner smooth muscle layer, from which they spread to the outer longitudinal smooth muscle layer, KCl was employed, as it depolarizes the cell membrane, resulting in Ca^2+^ influx by voltage-sensitive Ca^2+^ channels (Ca_V_) [25,26].

Our data showed that *S. platensis*, at 50, 150, and 500 mg/kg, reduced the ileum reactivity to KCl (Figure 1A, Table 1). On the other hand, supplementation reduced, in a dose-dependent manner, the ileum reactivity for CCh at 50 and 150 mg/kg, however, at 500 mg/kg, this effect was not observed (Figure 2A, Table 2). These data indicate that algae supplementation reduces the Ca^2+^ influx through Ca_V_ on smooth muscle or on myenteric nerves, which additionally could reduce the release of neurotransmitters, as ACh, explaining the reduced efficacy of the agonist in the animals supplemented with the algae. Furthermore, the contractile potency of KCl and CCh was increased, despite the reduced efficacy, at 50 and 500 mg/kg, respectively, since its pEC_50_ was increased at these doses. Since the increased potency of stimulation is associated by an augment in the number of receptors on cell membrane [27], this find can be explained by an increase expression of Ca_V_ and M_3_ (or a combination of both) promoted by the algae, probably as a compensatory mechanism for the reduced ACh release. However, the elucidation of the obtained results requires additional research; such statement can be confirmed by the examination of ACh levels in the ileum.

Physical exercise is characterized by removing the organism from homeostasis, due to the increased energy expenditure by the musculature, leading to physiological responses [28]. During exercise, the blood flow is diverted in greater quantities for the musculature and skin to oxygenate these tissues, leading to the splanchnic ischemia-reperfusion process. Because of this, these processes can generate reactive oxygen species, which disrupts normal cellular functioning due to the oxidative stress, which alters the function of macromolecules [29]. However, just as reactive oxygen species are generated, there is the antioxidant defense that fights these free radicals, and the body returns to homeostasis.

Some studies which have focused on the effects of strength training on organ functioning, especially vascular beds, have demonstrated vascular function improvement by strength exercise [30,31,32,33]. In addition, these studies showed a reduction on vascular reactivity by this modality of exercise [34]. However, concerning the gastrointestinal tract, little is known about the effects of physical exercise. The exercise promotes ischemia and motor changes in the intestine and intestinal mucosa [35], and shows that swimming as an acute and chronic aerobic exercise reduces ileum reactivity to both KCl and CCh [15,16]; however the influence of anaerobic exercise of resistance was not evaluated so far.

Then, we evaluated the influence of strength exercise on ileum reactivity and the combination with *S. platensis* supplementation, in view of a possible potentiation on the beneficial effects when associated; however, initially we confirmed the effectiveness of exercise by observing an increase in the time to perform the exercise sessions, and that the program of strength exercise with progressive loads does not promote an overtraining [36], observing that the activity of lactate dehydrogenase (LDH) and creatine kinase (CK) were not increased (data not showed).

We observed that the training decreased the reactivity of rat ileum to CCh, but not to KCl, indicating a preferential effect on ACh release than on Ca_V_ expression or activity. Additionally, when combined with the algae, there was a potentiation on the reduction on contractile reactivity, since this effect was observed at lower doses than occurred on sedentary rats (Figure 1 and Figure 2, Table 1 and Table 2), for both contractile agents. Therefore, these data reinforce a potentiation promoted by the algae on the effects of strength exercise on rat ileum contractility.

Despite these hypotheses, the data obtained do not allow an in-depth analysis of the mechanisms involved in these alterations, and additional studies are necessary to mechanistically elucidate these findings.

Studies of acute and chronic swimming exercise have shown that acute exercise does not promote oxidative stress in rat ileum. Furthermore, there is an increase after four weeks of exercise, but after six to eight weeks, oxidative stress is reduced, showing that the body underwent physiological changes to adapt to exercise and returned to homeostasis [15,16]. However, regarding the strength exercise, there are not data showing its effect on the balance oxidative stress and antioxidant defenses.

Literature data shows that *S. platensis* presents antioxidant activity due to the pigment phycocyanin, a very stable molecule and free radical scavenger [37], beyond its inhibitory effect on the production of superoxide anion [38,39]. In addition, other constituents contribute to the antioxidant benefits of the algae, as carotenoids, that regulate superoxide dismutase (SOD) and catalase (CAT), as well as block free radicals by chelation of metal ions, preventing lipid peroxidation; and vitamins B and E, that act as antioxidants via capture of radicals and metal chelating agents [40].

Our data showed that food supplementation with the algae, at the higher dose (500 mg/kg), decreased the level of oxidative stress in rat ileum, since there was a reduction in the MDA concentration measured; an oxidative damage marker formed by oxidation of lipid of cell membrane [41]. Differently, the strength training did not alter the formation of ROS, but the association of exercise and *S. platensis* supplementation reduced this parameter at 150 and 500 mg/kg (Figure 3), indicating that this combination promotes beneficial effect regarding the prevention of cell oxidative damage. Confirming these data, it was observed that both supplementation (500 mg/kg) and the association, at the same doses, improved the antioxidant capacity of rat ileum (Figure 4).

Taken together, these data point to a possible beneficial effect of food supplementation with *S. platensis*, as well as its association with strength training—this is preliminary data that needs further testing, including in humans to confirm possible effects such the reduction of oxidative damage in the intestine, which may contribute for a better intestinal functioning, especially during exposure to stress.

## 4. Material and Methods

### 4.1. Animals

Wistar rats (*Rattus norvegicus*), initially 2-months-old, weighing 200–300 g, were obtained from animal production unit of UFPB. The animals were kept under restricted food control with balanced diet (Labina^®^, São Paulo, Brazil), to avoid large differences in body weight and density, and had access to water ad libitum. They were maintained in rooms at 21 ± 1 °C and submitted to a 12 h light-dark cycle (light on from 6 to 18 h). Forty-eight hours after the last exercise session, the animals were fasted for 18 h (receiving only water *ad libitum* during this period), to avoid the influence of substances released during digestion, and then euthanized by cervical dislocation followed by cervical vessels section to proceed with the experimental analysis.

All experimental procedures were performed following the principles of animal care of the Guidelines for the ethical use of animals in applied etiology studies [42] and previously approved by UFPB Ethics Committee on Animal Use (Protocol/CEUA 0511/13).

### 4.2. Drugs

Calcium chloride bihydrate (CaCl_2_·2H_2_O), magnesium chloride hexahydrate (MgCl_2_·6H_2_O), and glucose (C_6_H_12_O_6_) were purchased from Vetec (Darmstadt, Germany). Sodium bicarbonate (NaHCO_3_) was purchased from Fmaia (Belo Horizonte, Brazil). Sodium chloride (NaCl) and potassium chloride (KCl) were purchased from Química Moderna (São Paulo, Brazil). Monosodium dihydrogen orthophosphate (NaH_2_PO_4_), sodium hydroxide (NaOH) and hydrochloric acid (HCl) were purchased from Nuclear (Brazil). These substances were employed in the physiological Tyrode solution for functional experiments. Carbamylcholine hydrochloride (CCh) was purchased from Merck (Darmstadt, Germany). Thiobarbituric acid, tetramethoxypropane and perchloric acid were purchased from Sigma-Aldrich (St. Louis, MO, USA). Carbogen mixture (95% O_2_ and 5% CO_2_) was obtained from White Martins (Danbury, CT, USA).

### 4.3. Spirulina Platensis

*S. platensis* was obtained as powder from the Dongtai Top Bio Engineering Co., Ltd. (Nanjing, China) (lot 20130320). A sample was analyzed for quality control by Farma Nostra laboratory (Anápolis, Goiás, Brazil) (Lot 1308771A) and handled by Dilecta pharmacy (João Pessoa, Paraíba, Brazil) (lot 20121025).

### 4.4. Experimental Protocol

Animals were divided into eight groups (five animals in each): Sedentary (S), trained (T), sedentary and supplemented with *S. platensis* at 50 (S50), 150 (S150), and 500 mg/kg (S500), and a combination of training and supplementation at 50 (T50), 150 (T150), and 500 mg/kg (T500). 

**Figure 5:**
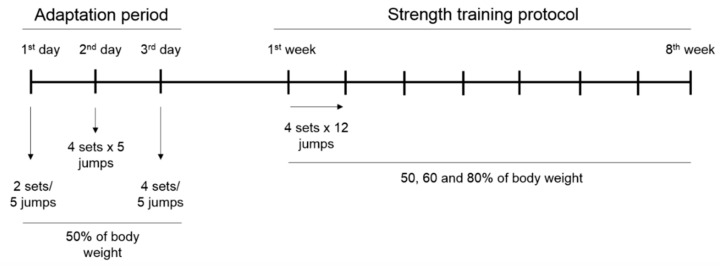
Strength training program. Adaptation period: 1–3 days; and strength training protocol: 1–8 weeks.

Animals of trained groups were submitted to a specific program of water jumping; for this, water was heated to around 32 °C [43]. The week of adaptation was consisted of three alternating sessions of exercise (1st day: 2 sets × 5 jumps, 2nd day: 4 sets × 5 jumps, and 3rd day: 4 sets × 5 jumps), with an overload of 50% of the body weight. The strength training program consisted of 4 sets of 12 repetitions, with a 30 second interval between sets; a progressive load was adjusted according to the animal weight, being 50% (1st and 2nd week), 60% (3th and 4th week), and 80% of body weight (5th to 8th week), anchored in the trunk of the animals by a vest. After 48 h from the end of the training, the animals were euthanized and the ileum was isolated (Figure 5).

For supplementation with *S. platensis,* the algae was dissolved in saline solution and orally administrated by gavage (volume: 2.5 mL/animal) along 8 weeks [44]. For trained + supplemented animals, administration was performed 30 min before exercise session and sedentary animals received saline solution [45].

### 4.5. Contractile Reactivity Measurement

Animals were euthanized. The ileum was immediately removed, cleaned of fat and connective tissue, and immersed in physiological solution at room temperature and bubbled with carbogen mixture. To register isotonic contractions, ileum segments (2–3 cm) were suspended by cotton yarn in organ bath (5 mL) and recorded on smoked drum through levers coupled to kymographs (DTF) under resting tension of 1.0 g at 37 °C [46]. The organ baths were warmed by a thermostatic pump Polystat 12,002 Cole-Palmer (Vernon Hills) and bathed by a Tyrode solution (pH 7.4) with the following composition (in mM): NaCl (150.0), KCl (2.7), CaCl_2_ (1.8), MgCl_2_ (2.0), NaHCO_3_ (12.0), NaH_2_PO_4_ (0.4), and d-glucose (5.5). After 30 min of stabilization period, an isotonic contraction was induced with KCl 30 mM, to verify the functionality of the organ, and 15 min after two similar cumulative concentration-response curves to KCl or CCh were obtained, as electro- and pharmaco-mechanical coupling contractile agents. The reactivity of ileum to that agents was assessed and compared between groups based on the values of maximum effect (*E*_max_) and negative logarithm of the concentration of contractile agents producing 50% of its maximal effect.

### 4.6. Lipid Peroxidation Assay

Lipid peroxidation in ileum was determined measuring the chromogenic product of 2-thiobarbituric acid (TBA) reaction with malondialdehyde (MDA), which is one of the products formed because of membrane lipid peroxidation [47]. Ileum segments from each animal were homogenized with KCl (1:1) and 250 μL of tissue homogenate were incubated at 37 °C for 60 min. Then, the mixture was precipitated with 35% perchloric acid and centrifuged at 1.207 *g* for 20 min at 4 °C. The supernatant was transferred to Eppendorf^®^ tubes and 400 μL of TBA 0.6% were added and incubated at 95–100 °C for 1 h. After cooling, the samples were read in spectrophotometer at wavelength of 532 nm. The results were expressed in μmol/L per gram of dry tissue.

### 4.7. Antioxidant Assay

Ileum segments from each animal were homogenized with KCl (1:1), centrifuged at 1.198× *g* for 10 min and 250 μL of supernatant were incubated at 37 °C for 60 min. Then, 100 μL of the tissue was added in an Eppendorf^®^ tube with 2 mL of 2,2-diphenyl-1-picrylhydrazyl (DPPH) solution (1.25 mL DPPH dissolved in 100 mL ethanol). The tubes were vortexed and allowed to stand for 30 min. They were centrifuged at 7.489× *g* at 20 °C for 15 min. Then, the samples were read in spectrophotometer at a wavelength of 515 nm. 

The results were expressed as a percentage of oxidation inhibition, following the equation:

AOA = 100 − [(DPPH-R)_S_/(DPPH-R)_W_ × 100]

where (DPPH-R)_S_ and (DPPH-R)_W_ correspond to the concentration of DPPH- remaining after 30 min, measured in the sample (S) and white (W) prepared with distilled water [48].

### 4.8. Statistical Analysis

Data were expressed as mean ± standard error of mean (S.E.M.). Cumulative concentration-response curves were fitted and pEC_50_ values were obtained by non-linear regression [28]. Multiple comparisons were performed by one- or two-way ANOVA followed by Tukey or Bonferroni’s post-test. The differences were considered significant when *p* < 0.05. All data were analyzed using GraphPad Prism^®^ software version 5.01 (GraphPad Software Inc., California, LA, USA).

## 5. Conclusions

An experimental model of smooth muscle is important for studying its functioning and new pharmacological and non-pharmacological strategies for the treatment of disorders, such as those affecting the intestinal tract. Our data point to a possible beneficial effect of food supplementation with *S. platensis*, as well as its association with strength training on oxidative stress and contractile reactivity. This is preliminary data needs further testing, including in humans, to confirm possible effects of the reduction of oxidative damage in the intestine, which may contribute for a better intestinal functioning, especially during exposure to stress.

## Figures and Tables

**Table 1:** Values of *E*_max_ (%) and pEC_50_ of KCl in the S, S50, S150, S500, T, T50, T150, and T500 groups in rat ileum.

KCl (M)	*E*_max_ (%)	pEC_50_
S	100.0	1.76 ± 0.02
S50	59.7 ± 5.8 * ^#^	2.04 ± 0.04 *
S150	71.6 ± 7.1 *	1.76 ± 0.04
S500	51.3 ± 4.5 * ^#^	1.63 ± 0.02
T	82.4 ± 7.5	1.78 ± 0.06
T50	14.0 ± 1.8 * ^#^^γ^	1.58 ± 0.09
T150	33.7 ± 5.7 * ^#^^γ^	1.65 ± 0.07
T500	83.3 ± 3.1	1.64 ± 0.04

Data are expressed as the mean and e.p.m (*n* = 3–5). One-way ANOVA followed by Tukey’s post-test: *E*_max_: * *p* < 0.05 (S vs. S50, S500, T50, and T150), ^#^ *p* < 0.05 (S50 and S500 vs. S150; T vs. T50 and T150), ^γ^ *p* < 0.05 (T50 and T150 vs. T500); pEC_50_: * *p* < 0.05 (S50 vs. S, S150 and S500).

**Table 2:** Values of *E*_max_ (%) and pEC_50_ of KCl in the C, C50, C150, C500, T, T50, T150, and T500 groups in rat ileum.

CCh (M)	*E*_max_ (%)	pEC_50_
S	100.0	5.99 ± 0.05
S50	92.5 ± 2.6	6.19 ± 0.11
S150	30.0 ± 0.9 *	6.26 ± 0.10
S500	53.3 ± 3.8 * ^#^	6.43 ± 0.15 *
T	57.1 ± 5.8 *	6.44 ± 0.14
T50	33.3 ± 2.0 * ^#^	6.35 ± 0.18
T150	56.6 ± 3.9 *	6.38 ± 0.20
T500	43.5 ± 4.5 *	6.07 ± 0.18

Data are expressed as the mean and e.p.m (*n* = 3–5). Two-way ANOVA followed by Bonferroni’s post-test: *E*_max_: * *p* < 0.05 (S or S50 vs. S150, and S500; S vs. T, T50, T150 and T500), ^#^ *p* < 0.05 (S150 vs. S500; T and T150 vs. T50); pEC_50_: * *p* < 0.05 (S vs. S500).

## Abbreviation List

Sedentary (S)
S + supplemented with algae at 50 (S50) 150 (S150)
S + supplemented with algae at 150 (S150)
S + supplemented with algae at 500 mg/kg (S500)
Trained (T)
T + supplemented (T50, T150, and T500)
2,2-diphenyl-1-picrylhydrazyl (DPPH)
Malondialdehyde (MDA)
Carbachol (CCh)
Potassium chloride (KCl)
Maximum effect (*E*_max_)
pEC_50_
Reactive oxygen species (ROS)
Acetylcholine (ACh)
Ca^2+^ channels (Ca_V)_
Lactate dehydrogenase (LDH)
Creatine kinase (CK)
Superoxide dismutase (SOD)
Catalase (CAT)
Calcium chloride bihydrate (CaCl_2_.2H_2_O)
Magnesium chloride hexahydrate (MgCl_2_.6H_2_O)
Glucose (C_6_H_12_O_6_)
Sodium bicarbonate (NaHCO_3_)
Sodium chloride (NaCl)
Monosodium dihydrogen orthophosphate (NaH_2_PO_4_)
Sodium hydroxide (NaOH)
Hydrochloric acid (HCl)
Carbamylcholine hydrochloride (CCh)
2-thiobarbituric acid (TBA)
